# Burden of Common Respiratory Pathogens Among Cats in China

**DOI:** 10.1002/vms3.70082

**Published:** 2024-11-22

**Authors:** Sajid Umar, Shaban Muhammad, Marwa mouzahim, Shelley Marie Pleva, Qiu Zhongqi, Yu Weidong, Di Gao

**Affiliations:** ^1^ Global Health Research Center (GHRC) Duke Kunshan University Kunshan China; ^2^ Division of Natural & Applied Sciences (DNAS) Duke Kunshan University Kunshan China; ^3^ Simba Pet Hospital (Tinglin Park branch) Kunshan China; ^4^ Play Pi Kangkang Pet Hospital Kunshan City Development Zone Kunshan China; ^5^ MSD Animal Health Shanghai Shanghai China

**Keywords:** cats, China, feline respiratory disease, prevalence

## Abstract

**Background:**

Feline respiratory disease complex (FRDC) is a set of illnesses which are primarily associated with different types of viruses and bacteria. There is scarcity of data on pathogens associated with FRDC in China.

**Objectives:**

The primary objective of this study was to investigate the prevalence and dynamics of FRDC pathogens in China.

**Methods:**

A total of 458 samples were retrieved from veterinary clinics during 2021–2024 from cats suffering from respiratory infections. Four viruses and three bacteria associated with FRDC were targeted for molecular detection with real time qPCR/RT‐qPCR assays.

**Results:**

At least 1 targeted respiratory pathogen was detected in 423 samples (92.3%), whereas no pathogens were detected in 7.7% of samples. Bacteria were detected in 32.1% of samples, and viruses were detected in 60.2% of samples. The prevalence of viruses detected were feline calicivirus (31.2%), feline herpesvirus‐1 (24.6%), influenza A virus (2.8%) and severe acute respiratory syndrome coronavirus‐2 (1.5%), whereas the detection rate for bacteria was *Mycoplasma felis* (15.5%), *Chlamydia felis* (10.2%) and *Bordetella bronchiseptica* (6.3%). Significantly higher cases were reported from kittens (57.4%). Pathogen detection was more common during the cold season. Mono‐infections involving one bacteria or virus were detected in 44.7% of samples, whereas coinfections were detected in 47.5% of samples. No quadruple coinfections were recorded in this study.

**Conclusions:**

The frequency of detection of feline respiratory pathogens alone or in combinations among diseased cats was high, indicating a heavy burden of respiratory infections among cats in Kunshan, China. Continued surveillance is desired, and newly emerged respiratory pathogens should also be monitored in routine diagnostic testing.

## Graphical abstract

Feline respiratory pathogens alone or in combinations were high, indicating a heavy burden of respiratory infections among cats. Mono‐infections involving one bacteria or virus were detected in 44.7% of samples, whereas coinfections were detected in 47.5% of samples. Veterinarians and researchers could benefit from these findings to develop better preventive strategies against feline respiratory pathogens.

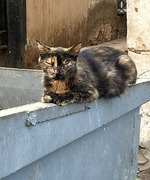

## 1 Introduction

Feline respiratory disease complex (FRDC), also known as cat flu, is a set of illnesses caused by different types of infectious agents, most commonly viruses but also bacteria, which affect upper respiratory tracts in cats. Viruses associated with FRDC include mainly feline calicivirus (FCV) and feline herpesvirus type 1 (FHV‐1). The main bacterial agents frequently detected in cats with FRDC include *Mycoplasma felis*, *Chlamydia felis* (formerly *Chlamydia psittaci* and *Chlamydophila felis*) and *Bordetella bronchiseptica* (Cohn 2011; Helps et al. 2005; Kim et al. 2024; Palombieri et al. 2022; Walter et al. 2020). Many of the pathogens involved in FRDC, such as FCV and FHV‐1, are highly contagious and can spread quickly in a shelter where many cats are housed in close proximity (Dinnage, Scarlett, and Richards 2009; Palombieri et al. 2022). FRDC may manifest as a range of symptoms, from mild respiratory discomfort to severe illness, and can be more pronounced in young kittens or immunocompromised cats (Sykes 2014). The cats show clinical signs of varying severity, including conjunctivitis, ocular discharge, sneezing, nasal discharge, fever, coughing and oral lesions (Cohn 2011). Multiple pathogens induce overlapping clinical signs, which are hard to differentiate among cats concurrently infected with more than one pathogen, making diagnosis and treatment really challenging for clinicians (Cohn 2011; Helps et al. 2005; Sykes 2014).

Coinfection with multiple respiratory pathogens is not uncommon in cats, especially those in high‐density populations such as shelters or catteries. These agents can affect similar tissues within the respiratory tract; a cat can be infected with more than one pathogen simultaneously. When cats are coinfected with more than one pathogen, it can complicate the clinical picture and may lead to more severe symptoms or prolonged illness. The stress associated with crowding and the close quarters itself can lead to increased shedding and transmission of these pathogens (Gao et al. 2023; Kim et al. 2024; Litster, Wu, and Leutenegger 2015; Liu et al. 2020; Nguyen et al. 2019; Palombieri et al. 2022). Influenza A viruses (IAVs) and severe acute respiratory syndrome coronavirus 2 (SARS‐CoV‐2) have indeed been identified in various animal species, including cats, highlighting the reverse zoonotic potential of these viruses, especially with cats living in proximity of birds, animals and humans (Palombieri et al. 2022). IAV cases in cats have been reported recently (Frymus et al. 2021; Wasik, Voorhees, and Parrish 2021). In the last two decades, five subtypes of IAV have been reported to cause acute respiratory illness in cats, including H5N1 (Keawcharoen et al. 2004; Kuiken et al. 2004; Leschnik et al. 2007; Marschall and Hartmann 2008; Rimmelzwaan et al. 2006; Thiry et al. 2007), H1N1 (Campagnolo et al. 2011; Fiorentini et al. 2011; Knight et al. 2016; Sponseller et al. 2010; Tangwangvivat et al. 2019), H5N6 (Cao et al. 2017; Lee et al. 2018; Yu et al. 2015), H7N2 (Blachere et al. 2018; Newbury et al. 2017) and H3N2 (Jeoung et al. 2013; Song et al. 2011). IAV‐infected cats exhibit varying clinical signs depending on the IAV subtype and occasionally lethal respiratory disease (Frymus et al. 2021; Kuiken et al. 2004). Furthermore, sporadic cases of SARS‐CoV‐2 have been reported in cats and several other animals during the COVID‐19 pandemic (Anderson et al. 2022; Cui et al. 2022; Palombieri et al. 2022; Thieulent et al. 2024). Beside domestic cats, wild feline species, such as pumas, tigers and lions, are also susceptible to SARS‐CoV‐2 natural infection. Cats suffering from SARS‐CoV‐2 infection can often present with similar clinical signs, making it difficult to distinguish among the various respiratory pathogens based on symptoms alone (Fritz et al. 2022; Palombieri et al. 2022; Thieulent et al. 2024). Keeping in mind the zoonotic potential of IAV and SARS‐CoV‐2, advanced rapid diagnostic assays are required to differentiate infection of these two viruses with other etiological agents of FRDC to implement appropriate public health measures and mitigate the threat of future pandemics.

Because respiratory diseases in cats and other animals can often present with similar clinical signs, it is difficult to distinguish among the various respiratory pathogens based on symptoms alone. Awareness of the possibility for coinfection is essential for veterinarians when considering diagnostic and treatment options. Therefore, sensitive and accurate diagnostic methods are essential for rapid identification of pathogens. Veterinary diagnostic platforms commonly use real‐time qPCR and RT‐qPCR assays to detect FRDC pathogens, which provide rapid accurate detection with high sensitivity (Thieulent et al. 2023; Thieulent et al. 2024). Commercial vaccines usually provide a good protein but cannot prevent the occurrence of FRDC. Even though vaccines may not completely prevent infection, they often reduce the severity and spread of disease within feline populations. The simultaneous presence of multiple pathogens may necessitate a broader therapeutic approach, such as the use of both antiviral medications to control a viral component and antibiotics to address secondary bacterial infections. Furthermore, supportive care measures are particularly important for cats with complex respiratory infections to maintain hydration and nutrition and to manage symptoms such as nasal congestion and conjunctivitis. Preventive measures like vaccination, stress reduction and good hygiene can help mitigate the risk of coinfection. Investigation of respiratory disease burdens in cats contributed to the prevention, diagnosis and treatment of cat infections. Although the prevalence of the pathogens associated with FRDC has been investigated worldwide, there are limited data on FRDC in cats in China. To the best of our knowledge, there is no published data from Kunshan city about FRDC, indicating that there is a need for epidemiological studies focusing on FRDC. This cross‐sectional study aims to provide important insights into the prevalence of various pathogens (e.g., SARS‐CoV‐2, IAV, FHV‐1, FCV, *M. felis*, *C. felis* and *B. bronchiseptica*) and dynamics of FRDC in this specific geographic region, which is critical for developing targeted intervention strategies.

## 2 Materials and Methods

### 2.1 Collection of Samples

This was a cross‐sectional epidemiological study which was performed on 458 samples (nasal, ocular, oropharyngeal swabs) collected from diseased cats exhibiting respiratory symptoms at various animal clinics in Kunshan from 2021 to 2024 (Figure S1). The cats which were exhibiting common respiratory clinical signs (ocular discharge, nasal discharge, sneezing and coughing) were included in this study. Nasal, ocular and oropharyngeal swabs from each cat were collected and pooled into a tube with universal transport media (Copan Diagnostics Inc, Italy). For each cat, the date of evaluation, approximate age, sex and associated clinical signs were recorded. However, information about breed, living environment, exposure history, vaccination status and treatment regimens was not recorded. The samples were stored at −80°C until tested for targeted pathogens. This non‐invasive study employing only swab sample collection did not require ethical approval from the Institutional Animal Care and Use Committee (IACUC). The entire process of sample collection from cats was carried out in accordance with international regulations and guidelines.

### 2.2 Nucleic Acid Extraction

Nucleic acid was extracted using 200 µL of pooled swab sample following the manufacturer's extraction protocol. Viral RNA/DNA Extraction Kit (Cat. #9766, Takara, Dalian, China) and Bacterial Genomic DNA Extraction Kit (Cat. #9763, Takara, Dalian, China) were used for the isolation and purification of nucleic acids (Fernandez et al. 2017; Kim et al. 2024). After extraction, nucleic acid samples were preserved at −80°C for subsequent analysis.

### 2.3 Molecular Detection of Respiratory Pathogens

For FCV, IAV and SARS‐CoV‐2, RNA within extracted samples was reverse transcribed using the PrimeScript RT Master Mix (CAT# RR036B, Takara, Dalian, China). Preliminary detection of respiratory pathogens was performed with real‐time qPCR/RT‐qPCR assays targeting four viruses (SARS‐CoV‐2, IAV, FCV and FHV‐1) and three bacteria (*M*. *felis, C*. *felis* and *B*. *bronchiseptica*) associated with FRDC (Helps et al. 2005; Lobova et al. 2019). Sequences of primers and probes have been described in Table S1. A two‐step RT‐qPCR Probe Kit (Takara, Dalian, China) was used for PCR amplifications by following specific thermocycling protocol on an MIC Real‐Time PCR system (Biomolecular Systems, Yatala, Australia) as described previously (Helps et al. 2005; Lobova et al. 2019; Thieulent et al. 2024). A specimen was considered positive if the cycle threshold (Ct) values obtained was below <40. All measures were taken to reduce contamination during PCR reactions. Moreover, all negative samples were re‐extracted and retested to confirm the findings of our diagnostic assays.

### 2.4 Data Analysis and Statistics

Excel spreadsheets were utilized for data entry. Seasonality was divided into two periods for analysis purposes: warm season (May to October) and cold season (November to April). Cats were grouped into kittens (<1 year old), adolescents (1–4 years old) and adults (>4 years old) for age‐related data analysis. Chi square and Fisher's tests were utilized to estimate the associations. A significant difference was depicted with a *p* value of <0.05. Animal and owner identification and sensitive information were removed before analysis to ensure confidentiality. Seasonal and age‐related variations in pathogen prevalence are pertinent for understanding the dynamics of FRDC in the local cat population.

## 3 Results

**FIGURE 1:**
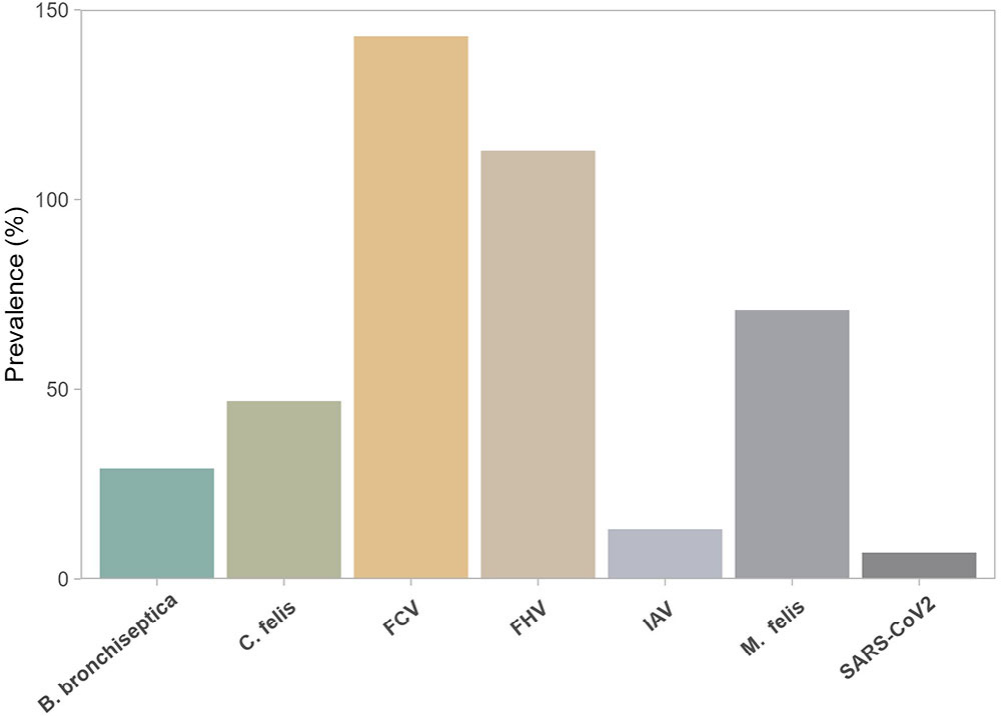
Overall prevalence of targeted respiratory pathogens. FCV, feline calicivirus; FHV, feline herpesvirus; IAV, influenza A virus.

Out of 458, at least 1 respiratory pathogen was detected in 423 samples (92.3%). Overall, 7.6% samples (35/458) did not reveal any examined pathogen in this study. Bacteria were detected in 32.1% (*n* = 147) of samples, and viruses were detected in 60.2% of samples (*n* = 276 out of 458 tested). Viral infections were the most prevalent among samples. The prevalence of viruses detected was FCV (31.2%), FHV‐1 (24.6%), IAV (2.8%) and SARS‐CoV‐2 (1.5%), whereas the detection rate for bacteria was *M. felis* (15.5%), *C. felis* (10.2%) and *B. bronchiseptica* (6.3%). As depicted in Figure 1 (Table S2), FCV (143/458 = 31.2%), FHV‐1 (113/458 = 24.6%) and *M. felis* (71/458 = 15.5%) were the most commonly detected pathogens, followed by *C. felis* (47/458 = 10.2%), *B. bronchiseptica* (29/458 = 6.3%), IAV (%, 13/458 = 2.8%) and SARS‐CoV‐2 (7/458 = 1.5%). Our findings showed that FCV, FHV‐1, *M. felis* and *C. felis* are the most prevalent pathogens linked with FRDC in cats. SARS‐CoV‐2 and IAV were the least detected pathogens, highlighting a lower prevalence or possibly less efficient transmission among cats.

**FIGURE 2:**
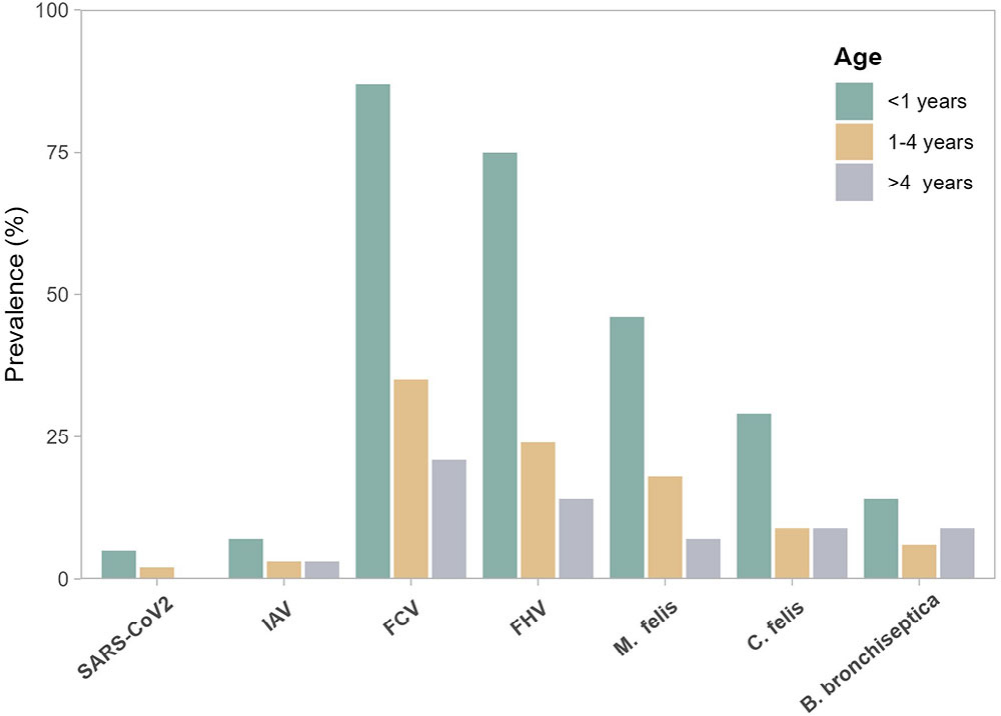
Pathogen prevalence by age group. FCV, feline calicivirus; FHV, feline herpesvirus; IAV, influenza A virus.

**FIGURE 3:**
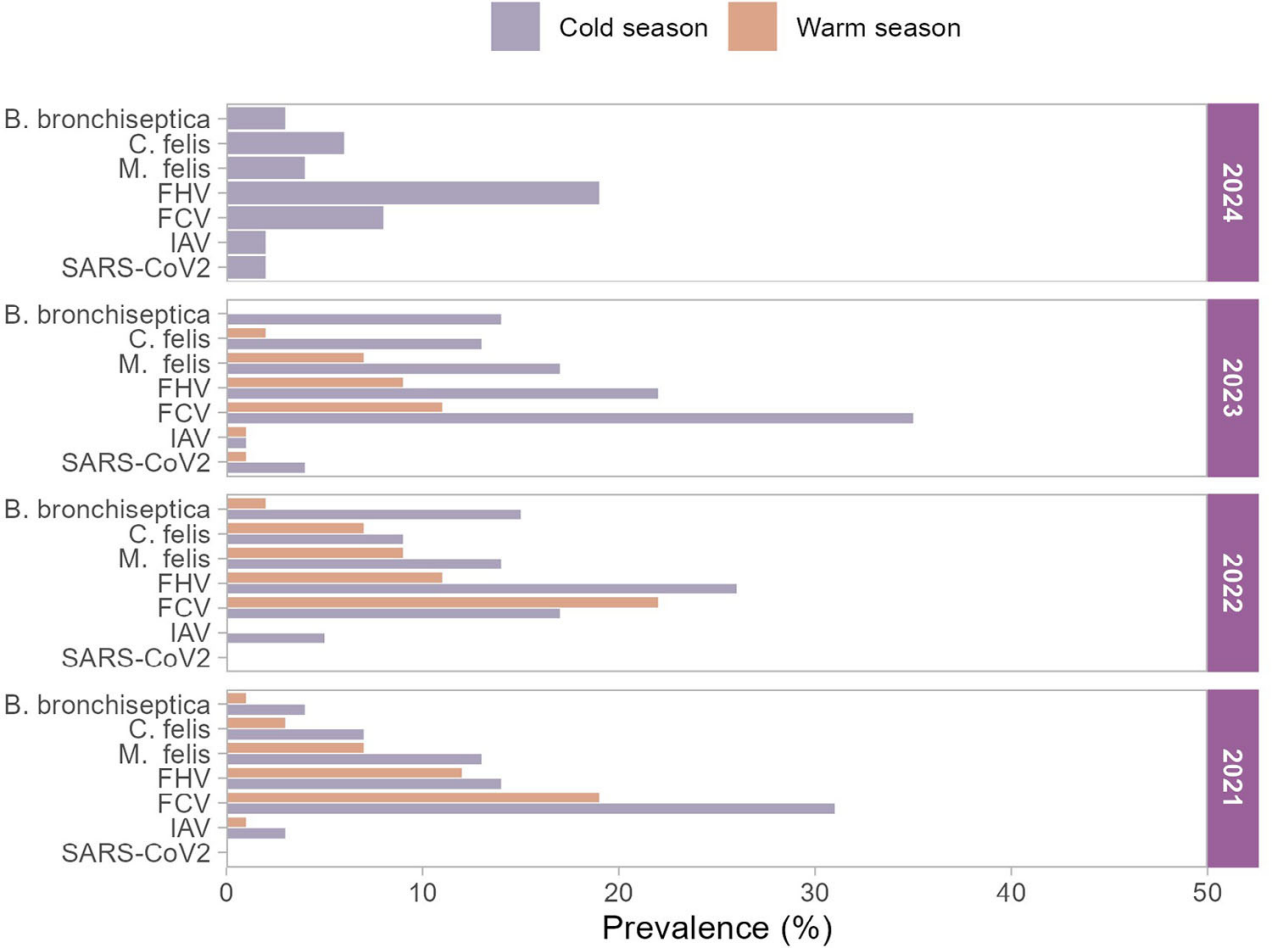
Prevalence of positives cases during cold and warm seasons. FCV, feline calicivirus; FHV, feline herpesvirus; IAV, influenza A virus.

Kittens (<1 year of age) were the most affected (263/458, 57.4%). Statistically significant differences were observed between age and frequency of FRDC pathogens (Figure 2, Table S3). SARS‐CoV‐2 was not detected among adult cats (>4 years). We also noticed the kittens were significantly more affected with FCV, FHV‐1 and *M. felis*. Adolescents (1–4 years) were the second most affected age group, and 97 cases (22.9%) were positive for FRDC pathogens. A total of 271 (59%) male (Tom) and 187 (41%) female cats (queens) were tested in the present study. Between sex and pathogen detection rate, a non‐significant association was observed (*p* > 0.05), although the detection rate of FRDC pathogens was higher in male cats. During this study, more samples were received during the cold season (*n* = 309) than in the warm season (*n* = 149). Cases of infected cats varied among different years and seasons, as illustrated in Figure 3 (Table S4). Pathogen detection was more common during cold season months. A significantly higher number of cats (*p* < 0.05) admitted to clinics with URTI during cold season than warm seasons of 2022 and 2023. Subsequently, the prevalence of positive cases also fluctuated year by year and was significantly higher during the cold season.

**FIGURE 4:**
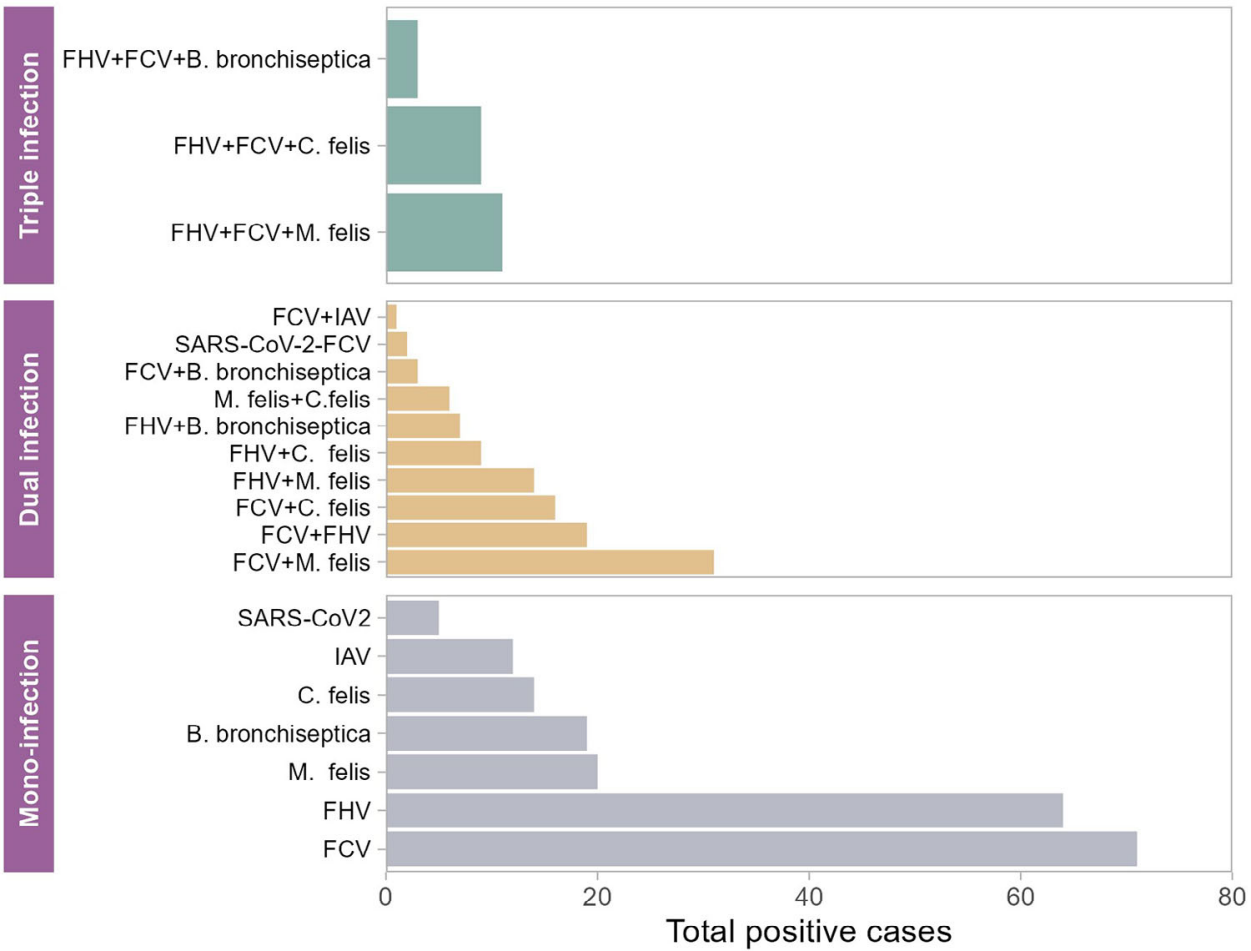
Detection rate of single agent infections and coinfections in the clinical samples. FCV, feline calicivirus; FHV, feline herpesvirus; IAV, influenza A virus.

Either one virus or one bacterium (mono‐infection) was detected in 44.7% of samples, whereas infections with more than one virus or bacteria (coinfections) were identified in 47.7% of samples (Figure 4, Table S5). Among the coinfections detected in this study, coinfection with FCV and *M. felis* was the most frequent (*n* = 31) followed by FCV + FHV‐1 (*n* = 19), FCV + *C. felis* (*n* = 16), FHV‐1 + *M. felis* (*n* = 14) and FHV‐1 + *C. felis* (*n* = 9). Coinfection with FHV‐1 and *B. bronchiseptica* was observed in seven cases, and coinfection with *M. felis* and *C. felis* was seen in six samples. IAV and SARS‐CoV‐2 had the least coinfections with other pathogens, and only two samples were coinfected with SARS‐CoV‐2 and FCV and one sample coinfected with IAV and FCV. Some notable coinfections involving three pathogens were also detected in study, which included: FHV‐1, FCV and *B. bronchiseptica* (*n* = 3); FHV‐1, FCV and *M. felis* (*n* = 11); and FHV‐1, FCV and *C. felis* (*n* = 9). A coinfection involving four pathogens (quadruple coinfections) was not detected in this study.

## 4 Discussion

FRDC or cat flu poses a significant health problem among cats worldwide. The present study observed the prevalence of four viruses (i.e., FCV, FHV‐1, IAV and SARS‐CoV‐2) and three bacteria (i.e., *B*. *bronchiseptica*, *M*. *felis* and *C*. *felis*). Association of respiratory pathogens with age, sex and seasonality was also explored. Overall prevalence of respiratory pathogens was higher in this study, which is consistent with previous studies from China and other regions of the world (Bannasch and Foley 2005; Di Martino et al. 2007; Dinnage, Scarlett, and Richards 2009; Fernandez et al. 2017; Gao et al. 2023; Hartmann et al. 2010; Nguyen et al. 2019; Thieulent et al. 2024; Walter et al. 2020). FCV was the most prevalent single pathogen (143/458; 31.2%) identified during 2021–2024, followed by FHV‐1 (113/458 = 24.6%), *M. felis* (71/458 = 15.5%), *C. felis* (47/458 = 10.2%), *B. bronchiseptica* (29/458 = 6.3%), IAV (13/458 = 2.8%) and SARS‐CoV‐2 (7/458 = 1.5%). Recently, Gao et al. (2023) reported a prevalence of 40.2%, 26.9%, 15.5% and 7.3% for FCV, *M. felis*, FHV‐1 and *C. felis* among cats in Wuhan, China. FCV and FHV‐1 are two non‐zoonotic viruses, the prevalence of which is extremely variable among cats (Bannasch and Foley 2005; Cohn 2011; Di Martino et al. 2007; Helps et al. 2005; Nguyen et al. 2019; Radford et al. 2007; Walter et al. 2020). A good vaccine programme can play a significant role in countering and managing FHV‐1 and FCV infections. However, despite regular vaccination, FRDC is still a serious health issue, especially for cats living in shelters (Nguyen et al. 2019). It has been reported that both non‐vaccinated cats (20%) and vaccinated cats (15%) can exhibit clinical signs associated with FRDC in a multi‐cat living (Bannasch and Foley 2005; Binns et al. 2000; Fernandez et al. 2017; Helps et al. 2005; Nguyen et al. 2019; Sykes 2014). Both viruses are shed by infected cats throughout life and are very resistant to harsh environmental conditions, which makes viruses stable and persist for months in the environment (Nguyen et al. 2019; Radford et al. 2007). Occasionally, a mutated form of FCV can occur that is highly virulent and can cause much more severe, systemic disease (Radford et al. 2007; Walter et al. 2020).

*M*. *felis* was the most common bacterial agent detected in this study (15.5%), which agrees with previous studies (Fernandez et al. 2017; Litster, Wu, and Leutenegger 2015; Thieulent et al. 2024). It is speculated that *M. felis* may assist other pathogens in FRDC by inducing pathological damage to the respiratory tissue of cats. A prevalence of 10.2% and 6.33% were observed for *C. felis* and *B*. *bronchiseptica*, respectively. *C. felis* has been reported to have a prevalence of less than 10% (Bannasch and Foley 2005; Cohn 2011; Di Martino et al. 2007; Helps et al. 2005). *B. bronchiseptica*, which was recently implicated as having a significant role in FRDC, has a reported prevalence of less than 15% (Bannasch and Foley 2005; Cohn 2011; Di Martino et al. 2007; Helps et al. 2005; Walter et al. 2020), although the seroprevalence in some studies has been found to be much higher (Helps et al. 2005).

During this study, we detected human IAV (H1N1, H3N2) and SARS‐CoV‐2 among cats. Domestic cat population has increased rapidly worldwide during the 21st century. There are approximately 65 million domestic cats in mainland China, which usually have close contact with their owners. These close contacts create more chances for pathogen spillover among humans and cats, which could lead to the emergence of new pathogenic strains or variants. Due to a close association between humans and companion animals, there is huge risk of reverse zoonotic events. Recently, Umar et al. reported reverse zoonosis events of human seasonal IAVs among cats in mainland China. Natural infections among cats with H1N1 (Campagnolo et al. 2011; Fiorentini et al. 2011; Knight et al. 2016; Sponseller et al. 2010; Tangwangvivat et al. 2019) and H3N2 (Jeoung et al. 2013; Song et al. 2011) have been reported. Moreover, sporadic natural infection of SARS‐CoV‐2 among domestic and wild cats was reported during the COVID‐19 pandemic (Anderson et al. 2022; Cui et al. 2022; Fritz et al. 2022; Palombieri et al. 2022; Thieulent et al. 2024). The non‐detection of SARS‐CoV‐2 in adult cats may point towards an age‐related defence mechanism or differing behavioural patterns reducing exposure risk.

Clinical signs among positive cats were variable and included oral lesions, sneezing, coughing and nasal and ocular discharge. Differential diagnosis was a challenge because clinical association with different pathogens were similar in this study. However, FHV‐1 and FCV infections were associated with pronounced eye and oral lesions, respectively. Given the variability in clinical signs, this finding is consistent with as observed in previous studies (Bannasch and Foley 2005; Binns et al. 2000; Cohn 2011; Fernandez et al. 2017; Gao et al. 2023; Kim et al. 2024; Nguyen et al. 2019). Kittens (<1 years of age) were at greater risk for infections, suggesting that age is an important factor in susceptibility to FRDC pathogens. A previous study had found that younger cats were commonly infected, and they tended to develop more severe clinical signs (Cohn 2011; Dinnage, Scarlett, and Richards 2009; Fernandez et al. 2017; Gao et al. 2023; Kim et al. 2024; Nguyen et al. 2019). This could be due to several reasons, including undeveloped immunity, increased stress, increased exposure and biological factors inherent to kittens (Dinnage, Scarlett, and Richards 2009; Fernandez et al. 2017; Gao et al. 2023; Nguyen et al. 2019; Palombieri et al. 2022). The nature of the causative agent, nutritional status and living conditions should also be considered to better understand this pattern.

There was no statistically significant relationship found between the sex of the cats and pathogen occurrence, even though a higher detection rate was observed in male cats. These findings are consistent with another study, which reported no association between gender and pathogen prevalence among sheltered cats was noted (Bannasch and Foley 2005). In contrast to our findings, a significantly higher detection rate was observed among male cats in a previously conducted study (Binns et al. 2000). The higher incidence rate among males could be linked to their more activities outside their residence, which gives more opportunity to catch pathogens from the environment spending (Dinnage, Scarlett, and Richards 2009; Gao et al. 2023). A significant seasonal pattern was noticed, with more infected cases in colder seasons. The infection rate of FCV and FHV‐1 was the highest in winter (63.6% and 71.6%, respectively). Significantly, higher cases during the cold season are consistent with the findings of a study in Wuhan, China (Gao et al. 2023). This could be related to the closer confinement of cats indoors during cold weather, which facilitates the spread of infectious agents. The seasonal fluctuations also underscore potential environmental and behavioural factors that contribute to the spread of respiratory infections. A significantly higher number of cases during cold seasons could influence clinical awareness and preparedness for respiratory outbreaks in colder months.

A higher coinfection rate was recorded among cats, which could potentially exacerbate clinical signs and complicate treatment options. Coinfection among FCV, FHV‐1 and *M. felis* was the most frequent. IAV and SARS‐CoV‐2 had the least coinfections with other pathogens, and only two samples were coinfected with SARS‐CoV‐2 and FCV and one sample coinfected with IAV and FCV. Coinfection with multiple respiratory pathogens is not uncommon in cats, especially those in high‐density populations such as shelters or catteries. When cats are coinfected with more than one pathogen, it can complicate the clinical picture and may lead to more severe symptoms or prolonged illness. The stress associated with crowding and the close quarters itself can lead to increased shedding and transmission of these pathogens. Coinfection can exacerbate clinical signs and make diagnosis more challenging for veterinarian (Palombieri et al. 2022; Sykes 2014). Surprisingly, no cases of quadruple infections were observed in this study, which might be partly due to the relatively lower prevalence of some pathogens like SARS‐CoV‐2 and IAV or could be a function of the sample size and the populations sampled. Neither a virus nor bacteria were detected in 7.6% tested samples (35/458) examined during the study period. According to veterinarian information, all cats sampled in this study were showing clinical signs in this study. Potential issues with sensitivity of detection with our diagnostic assays, inadequate sample collection or handling, or the presence of a pathogen(s) other than those we tested for (e.g., fungal pathogens) could be possible explanations. In addition, some non‐infectious diseases, such as autoimmune diseases (e.g., allergy), neoplasia, foreign bodies and chronic rhinosinusitis, may also produce respiratory distress among cats (Hartmann et al. 2010; Nguyen et al. 2019). We did not test fungal pathogens in these samples. It would be interesting to know the prevalence and coinfection status of fungal pathogens. Additionally, the preservation of samples at −80°C for a long time could have reduced sensitivity PCR detection. Furthermore, advances in technology have led to the discovery of some novel pathogens among cats in recent years. Hence, their role in the development of FRDC cannot be ignored.

Our study had a few limitations. We could not collect data about breed, vaccination status and residential density of cats in this present study, which could have made the results more interesting and valuable. Our sampling area represents a small geographical area within Jiangsu province, China; therefore, a conclusive regional pattern could not be drawn. Nevertheless, our study provided a comprehensive investigation on feline respiratory pathogens and demonstrated that pathogens associated with FRDC are frequent despite regular vaccination programme in Kunshan, China. These results might be suggesting that there is a need to develop new multivalent vaccines, including emerging pathogens along with feline core vaccines. The cat flu multivalent vaccines are not available in combination with *B. bronchiseptica*, *M. felis* and *C. felis*. Vaccination is a cornerstone of infectious disease control. Through vaccination, the spread of pathogens is reduced, and the severity of diseases is often lessened. Therefore, having more inclusive vaccines could be crucial to prevent the pathology. Client education about FRDC and awareness campaigns about vaccination programmes could decrease the prevalence of FRDC pathogens.

## 5 Conclusions

Our study provides baseline epidemiological data on the burden of respiratory pathogens in cats in China, which could help policy makers and veterinarians to develop better prevention strategies. We suggest some recommendations for better control and prevention of FRDC in future. First, pathogens are continuously evolving, and new pathogen detection at a rapid rate with advanced molecular technologies makes FRDC diagnosis more complex and challenging; therefore, more sophisticated diagnostic assays are desired. Second, given the number of samples found negative for all tested pathogens in this study, we suggest evaluating samples for other novel pathogens, including *Pasteurella multocida*, *Aspergillus fumigatus*, *Moraxella catarrhalis* and *Pneumocystis carinii*, to explore their role in FRDC. Finally, a continued surveillance programme to monitor FRDC pathogens and bringing more inclusive vaccines are critical for overall disease management in cats.

## Author Contributions

**S.U**.: conceptualization. **S.U**., **Q.Z**., **Y.W**. and **D.G**.: methodology. **S.U**.: validation. **S.M**., **M.M**., **S.M.P**. and **S.U**.: formal analysis. **S.U**., **Q.Z**., **Y.W**. and **D.G**.: investigation. **S.U**.: resources. **S.U**., **M.M**. and **S.M.P**.: data curation. **S.U**., **M.M**. and **S.M.P**.: writing—original draft preparation. **S.U**.: writing—review and editing. **S.U**.: visualization. **S.U**.: supervision. **S.U**.: project administration. **S.U**.: funding acquisition.

## Ethics Statement

The authors have nothing to report.

## Conflicts of Interest

The authors declare no conflicts of interest.

### Peer Review

The peer review history for this article is available at https://publons.com/publon/10.1002/vms3.70082.

## Supporting information



Supporting information

Supporting information

## Data Availability

The data that support the findings of this study are available on request.
