# Supplementary figures and images for: Burden of Common Respiratory Pathogens Among Cats in China
Source: Vet Med Sci. 2024 Nov 22;11(1):e70082. doi: 10.1002/vms3.70082 (PMC11582471; doi:10.1002/vms3.70082)

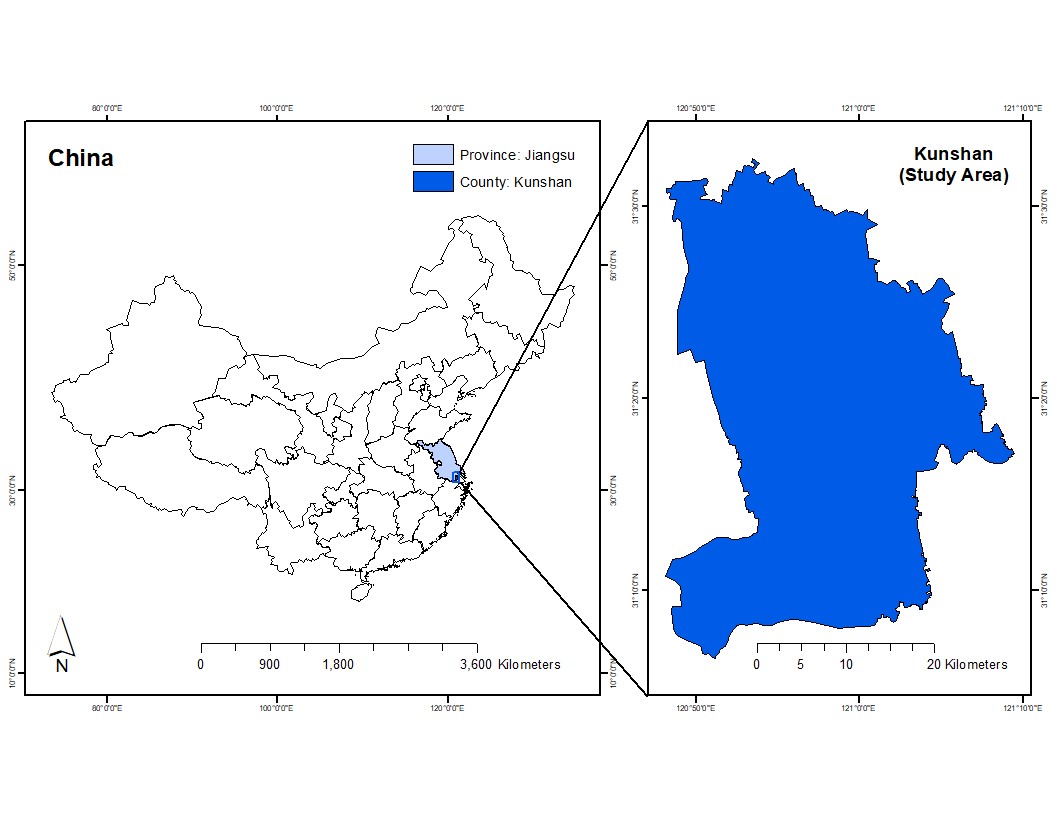

Supplement: Supplementary file 1 — Supporting information [file VMS3-11-e70082-s002.jpeg]
