# Supplementary material for: Burden of Common Respiratory Pathogens Among Cats in China
Source: Vet Med Sci. 2024 Nov 22;11(1):e70082. doi: 10.1002/vms3.70082 (PMC11582471; doi:10.1002/vms3.70082)
Supplement: Supplementary file 2 — Supporting information [file VMS3-11-e70082-s001.docx]

**Supplementary Table 1:** List of primers and thermocycling condition which were used in this study to detect seven respiratory pathogens.

| **Target gene** | **Primer name** | **Primers and probe sequences (5′-3′)** | **Nucleotide position** | **Product size (bp)** | **GenBank ID** | **Reference** |
| --- | --- | --- | --- | --- | --- | --- |
| SARS-CoV-2  (Nucleocapsid gene) | SARS-CoV2-F | GACCCCAAAATCAGCGAAAT | 28,287–28,306 | 72 | MN985325.1 | [39] |
|  | SARS-CoV2-R | TCTGGTTACTGCCAGTTGAATCTG | 28,358–28,335 |  |  |  |
|  | SARS-CoV2-P | **FAM**-ACCCCGCATTACGTTTGGTGGACC-**BHQ1** | 28,309–28,332 |  |  |  |
| Influenza A virus (Matrix gene) | IAV-M-F | AGATGAG**Y**CTTCTAACCGAGGTCG | 24–47 | 101 | MF978391.1 | [39] |
|  | IAV-M-R | TGCAAAGACATCTTCAAGTCTCTG | 124–101 |  |  |  |
|  | IAV-M-P | **FAM-**TCAGGCCCCCTCAAAGCCGA- **BHQ1** | 74–93 |  |  |  |
| Feline calicivirus (ORF-1) | FCV-F | GTTGGATGAACTACCCGCCAATC | 2413-2435 | 121 | M86379 | [17] |
|  | FCV-R | CATATGCGGCTCTGATGGCTTGAAACTG | 2534-2507 |  |  |  |
|  | FCV-P | **FAM-**TCGGTGTTTGATTTGGCCTG **BHQ1** | 2456-2475 |  |  |  |
| Feline herpesvirus 1  (TK gene) | FHV-F | GGACAGCATAAAAGCGATTG | 173-192 | 74 | M26660 | [17] |
|  | FHV-R | AACGTGAACAACGACGCAG | 247-229 |  |  |  |
|  | FHV-P | **FAM-**AATTCCAGCCCGGAGCCTCAAT **BHQ1** | 201-222 |  |  |  |
| *Mycoplasma felis* (16sRNA) | MF-F | CTACGGAGACAACTGCCTAAG | 53–73 | 103 | AY274256.1 | [27] |
|  | MF-R | GTACTCCGTAG-AAAGGAGGTAATC | 133–156 |  |  |  |
|  | MF-P | **FAM-**ACAAGGTATCCCTACGAGAACG-TGGG-**BHQ1** | 103–128 |  |  |  |
| *Chlamydia felis* (ompA gene) | CF-F | GCTAGGAGCGTCTAATGGTTAC | 387–408 | 77 | KP165540.1 | [27] |
|  | CF-R | GTTCCTGCAA-GACCAATCAATC | 443–464 |  |  |  |
|  | CF-P | **FAM**-AGCAAGTTCTGATGCATTTAACC-TTGTCG- **BHQ1** | 414–442 |  |  |  |
| *Bordetella bronchiseptica*  (FhaB gene) | BB-F | GGAACTTCAAC-TCCACGTATGA | 821–842 | 92 | AF140678.1 | [27] |
|  | BB-R | ATTCGGTGTAGGTCGGATAGA | 893–913 |  |  |  |
|  | BB-P | **FAM-**TGG-ACCAGAATCGCTGGGAATACA-**BHQ1** | 860–883 |  |  |  |

**Supplementary Table 2:** Overall prevalence of targeted respiratory pathogens.

| **Pathogens** | **No. of positives (n = 423/458)*** | **% positive (92.3%)*** |
| --- | --- | --- |
| SARS-CoV-2 | 7 | 1.52 |
| IAV | 13 | 2.83 |
| FCV | 143 | 31.2 |
| FHV-1 | 113 | 24.6 |
| *M.felis* | 71 | 15.5 |
| *C.felis* | 47 | 10.2 |
| *B.bronchiseptica* | 29 | 6.33 |

*Positive samples for at least one targeted pathogen, 35/458 (7.6%) specimens were negative for the targeted pathogens tested. IAV= Influenza A virus, SARS-CoV-2= Severe acute respiratory syndrome coronavirus-2, FCV= Feline calicivirus, FHV-1= Feline herpesvirus type 1, *M.felis= mycoplasma felis, C.felis= Chlamydia felis , B.bronchiseptica= Bordetella bronchiseptica.*

**Supplementary Table 3**: Pathogen prevalence by age group (n, %)

| **Targeted Pathogen** | **<1 years**  (*n* = 271) | **1-4 years**  (*n* = 103) | **>4 years**  (*n* = 83) | ***P value*** |
| --- | --- | --- | --- | --- |
| SARS-CoV-2 | 5 (1.84) | 2 (1.9) | 0 (0) | 0.453 |
| IAV | 7 (2.5) | 3 (2.9) | 3 (3.6) | 0.884 |
| FCV | 87 (32.1) | 35 (33.9) | 21 (25.3) | 0.404 |
| FHV-1 | 75 (27.6) | 24 (23.3) | 14 (16.8) | 0.127 |
| *M. felis* | 46 (16.9) | 18 (17.4) | 7 (8.4) | 0.141 |
| *C. felis* | 29 (10.7) | 9 (8.7) | 9 (10.8) | 0.841 |
| *B. bronchiseptica* | 14 (5.1) | 6 (5.8) | 9 (10.8) | 0.173 |

**Supplementary Table 4**: Prevalence of positives cases during cold and warm seasons.

| Year | Seasons* | Total sample | Total positives | No. of Positive Cats/Tested Cats (%) for each respiratory pathogen | | | | | | |
| --- | --- | --- | --- | --- | --- | --- | --- | --- | --- | --- |
|  |  |  |  | SARS-CoV2 | IAV | FCV | FHV | *M. felis* | *C. felis* | *B*.*bronchiseptica* |
| 2021 | Cold season | 73 | 72 | 0 | 3 | 31 | 14 | 13 | 7 | 4 |
|  | Warm season | 46 | 43 | 0 | 1 | 19 | 12 | 7 | 3 | 1 |
|  |  | *P value* | 0.3191 | 0.961 | 1.000 | 0.509 | 0.907 | 0.804 | 0.685 | 0.961 |
| 2022 | Cold season | 89 | 86 | 0 | 5 | 17 | 26 | 14 | 9 | 15 |
|  | Warm season | 59 | 51 | 0 | 0 | 22 | 11 | 9 | 7 | 2 |
|  |  | *P value* | 0.0462 | - | 0.165 | 0.023 | 0.208 | 1.000 | 0.948 | 0.024 |
| 2023 | Cold season | 98 | 96 | 4 | 1 | 35 | 22 | 17 | 13 | 4 |
|  | Warm season | 44 | 31 | 1 | 1 | 11 | 9 | 7 | 2 | 0 |
|  |  | *P value* | 0.0000035 | - | 1.000 | 0.286 | 0.963 | 1.000 | 0.205 | 0.417 |
| 2024* | Cold season | 49 | 44 | 2 | 2 | 8 | 19 | 4 | 6 | 3 |
| Overall prevalence | | 458 | 423 (92.3%) | 7  (1.52%) | 13 (2.83%) | 143 (31.2%) | 113 (24.6) | 71 (15.5%) | 47 (10.2%) | 29  (6.33) |

Warm season (May to October), cold season (November to April).* Samples were collected from January to April in 2024 and covered most of the cold season.

**Supplementary Table 5:** Detection rate of single agent infections and co-infections in the clinical samples.

| **Pathogens** | **No. of Positive samples** | **% of Positive samples** |
| --- | --- | --- |
| ***Respiratory infections associated with one pathogen*** | **205/458*** | **44.7** |
| SARS-CoV-2 | 5 | 1.09 |
| IAV | 12 | 2.6 |
| FCV | 71 | 15.5 |
| FHV | 64 | 14.0 |
| *M.felis* | 20 | 4.4 |
| *C.felis* | 14 | 3.0 |
| *B.bronchiseptica* | 19 | 4.1 |
| ***Respiratory infections associated with two pathogens*** | **102/458*** | **22.3** |
| SARS-CoV-2+ FCV | 2 | 0.43 |
| FCV+FHV | 19 | 4.1 |
| FHV+M.felis | 14 | 3.0 |
| FCV+M.felis | 31 | 6.8 |
| FHV+C.felis | 9 | 2.0 |
| FCV+C.felis | 16 | 3.5 |
| FCV+IAV | 1 | 0.2 |
| FCV+ B.bronchiseptica | 3 | 0.6 |
| FHV+ B.bronchiseptica | 7 | 1.5 |
| ***Respiratory infections associated with three pathogens*** | **23/458*** | **5.02** |
| FHV+FCV+B.bronchiseptica | 3 | 0.6 |
| FHV+FCV+M.felis | 11 | 2.4 |
| FHV+FCV+C.felis | 9 | 2.0 |

*35/458 (7.6%) specimens were negative for the targeted pathogens tested.
